# Note on Intra-Uterine Medication and Sterility

**Published:** 1879-03

**Authors:** W. S. Playfair


### Selections.

### NOTE ON INTRA-UTERINE MEDICATION AND STERILITY.

By W. S. PLAYFAIR, M.D., F.R.C.P.

I have been much interested by Mr. Wiglesworth's case of occlusion of the os and cervix uteri produced by the application of fuming nitric acid, published in the last number of the Obstetrical Journal. It certainly teaches a lesson of caution in the use of that remedy which is not needless, since it has been applied by some, especially in Ireland, with a freedom which has always seemed to me somewhat rash. My own experience with regard to it is not very great. I have, however, used it in many cases of severe menorrhagia, associated with endo-metritis, frequently with veiy remarkable benefit, and, I am bound to say, without ever having seen any ill effects follow. My object, however, in writing this note is to comment on the latter part of Mr. Wiglesworth's paper, in which he propounds the theory that the application of caustics to the interior of the uterus may be followed by sterility. If this were the case it would, no doubt, be a strong objection to their use. It would be interesting to hear the experience of others on this point. So far as my own goes it is directly opposed to Mr. Wiglesworth's. I may claim to have paid a good deal of attention to this matter, having communicated to the meeting of the British Medical Association, at Leeds, in 1869, a paper on the treatment of chronic uterine catarrh, which I believe was the first in this country in which systematic intra-uterine medication was advocated, and subsequently a series of lectures on "Intra-Uterine Medication in the Treatment of Chronic Uterine Catarrh." For the past ten years scarcely a day has passed on which I have not practiced intra-uterine medication in public or private practice, possibly riding my own hobby a little hard, as many of us are apt to do. Not only have I never seen anything in the least approaching to Mr. Wiglesworth's case, but very rarely indeed anything beyond the merest transitory irritation. Indeed, I am as sure as I can be of any fact in medicine, that there are a large class of otherwise intractable cases, which yield, I do not say easily, but certainly, to properly conducted treatment of this kind. So far from having any reason for thinking that it tends to produce sterility, my own experience would lead to the very opposite conclusion. It has been a matter of every day experience with me to meet with cases of chronic endometritis, with sub-involution, after a labor, perhaps years before, in which intra-uterine medication has been followed so rapidly by impregnation, as to leave no doubt of the result being due to the removal of the cause which led to sterility. In cases of this kind there is generally a large, bulky and possibly flexed uterus, with an abraded cervix, a patulous cervical canal, and much glairy mucus pouring from it. So commonly have I found pregnancy followed the cure of these conditions, that I have over and over again remarked to patients, who have expressed themselves as being rather aggrieved at finding themselves in the family way, that I had come to consider pregnancy as the nearly certain result and proof of a satisfactory cure. Only this afternoon, I happened to see a patient with Mr. Tait, of Highbury, whose case affords a good illustration of this. She had been a great sufferer from conditions very similar to those mentioned above, for four or five years, during which she never became pregnant. A few months ago she was treated by intra-uterine medication with carbolic acid with marked benefit, and now she is undoubtedly pregnant.

It may well be asked, why should such treatment produce sterilty? It is, no doubt, very desirable that we should possess accurate information, which we do not now do, as to the state of the uterine mucous membrane in such cases, and the caustics upon it. Pending the acquisition of such knowledge, I think it fair to assume that the result of such application is similar to that on the mucous membrane covering the cervix, and this is open to our inspection. Any one can satisfy himself that after swabbing a florid, abraded, granular, and bleeding cervix several times with a suitable application, it assumes the smooth, velvety appearance it ought to have in the healthy state. What reason is there to doubt that something similar occurs in the uterine mucous membrane, since its treatment is generally followed not only by the relief of pain and other local symptoms, but by healthy menstruation and the arrest of the glairy mucous discharge so characteristic of its morbid state? Nor, theoretically, is it at all difficult to understand why abundant catarrhal discharge from the uterus should prevent impregnation. Unless, therefore, some more valid evidence of intra-uterine medication producing sterility is brought forward than Mr. Wiglesworth produces, I shall continue to hold the opinion I had formed, that in a large proportion of cases of chronic endo-metritis and uterine catarrh it is one of the best means of removing it.

I do not enter into the relative merits of the various applications to be used, although I venture to quote a passage from my lectures on this subject to show why, as I still believe, carbolic acid is the best, and free from the risk of producing the result, which followed the use of nitric acid in Mr. Wiglesworth's case. "There are certain properties, too, possessed by carbolic acid, which render it preferable to all others as an intra-uterine application. Neumann, of Vienna, has shown that when applied in a tolerably concentrated form, such as I use, it causes the tissues to shrink and mummify, but they never swell; nor does it produce an eschar, as do the stronger caustics, such as potassa fusa, the acid nitrate of mercury, or even nitrate of silver. We can, therefore, use it freely without any risk of producing contraction of the canal of the cervix—a result which has followed the use of other agents. Certainly no case has come under my observation where the slightest approach to such a result has followed the use of carbolic acid.

Although foreign to the immediate object of this note, I may take this opportunity of saying that increased experience has taught me that intra-uterine medication is best practised within the ten days immediately following the cessation of a period, probably because the deeper layers of the uterine membrane are then reached in consequence of their denudation during menstruation. If practised towards the end of the menstrual interval it is sometimes apt to bring on menstruation prematurely. Practically, I find that two applications, at an interval of three or four days from each other, during the time I have indicated, are all that is required.

